# Mapping Life Satisfaction Over the First Years of Cohabitation Among Former Singles Living Alone in UK and Germany

**DOI:** 10.1111/jopy.70013

**Published:** 2025-08-18

**Authors:** Usama EL‐Awad, Robert Eves, Justin Hachenberger, Theresa M. Entringer, Robin Goodwin, Anu Realo, Sakari Lemola

**Affiliations:** ^1^ Faculty of Psychology and Sports Sciences Bielefeld University Bielefeld Germany; ^2^ Department of Psychology, Lifespan Health and Wellbeing Group University of Warwick Coventry UK; ^3^ Institute of Psychology University of Greifswald Greifswald Germany; ^4^ German Socio‐Economic Panel German Institute for Economic Research (DIW Berlin) Berlin Germany; ^5^ Department of Psychology University of Warwick Coventry UK; ^6^ School of Educational Sciences Tallinn University Tallinn Estonia; ^7^ Institute of Psychology University of Tartu Tartu Estonia

**Keywords:** cohabitation, life satisfaction, marriage, singles living alone

## Abstract

**Objective:**

As social norms and relationship dynamics evolve, it is important to examine how transitions from singlehood to partnership, cohabitation, and marriage relate to well‐being.

**Method:**

Using data from two large panel studies in the UK and Germany (1984–2019), we identified *N* = 27,459 individuals who reported being single and living alone at least once. Analyses focused on a subset (*N* = 1103; M_age_ = 38.35, SD_age_ = 13.87; 43.8% women) who later entered a relationship and moved in with a partner.

**Results:**

Life satisfaction increased over the short to medium term after cohabitation across most socio‐demographic groups. The increase peaked in the year of moving in (Δ ≈ 0.48 SD) and remained above pre‐transition levels for the 2 subsequent years analyzed. Those who had found a partner one year before had already achieved significantly higher life satisfaction, while cohabitation showed no additional effect. Marriage showed a short‐lived additional effect in the early 1990s, but not more recently. Lower‐income individuals experienced a stronger post‐peak decline.

**Conclusion:**

Findings suggest that well‐being increases are more closely aligned with relationship formation than with cohabitation or marriage. Among participants already in a relationship, increases in well‐being were observed prior to cohabitation, suggesting anticipatory effects.

The need for love and belonging is fundamental to humans (Maslow 1958). Living in a romantic relationship is an important part of fulfilling this need (Pinkus 2020) and an important life goal for many individuals (Skaletz and Seiffge‐Krenke 2010). Adults in a relationship likely enjoy better health and greater longevity (Holt‐Lunstad et al. 2008), higher social support, and life satisfaction (Vaillant 2012). Life satisfaction—referring to an individual's overall evaluation of their life as a whole—has been proven to be a key predictor of broad mental health (Fergusson et al. 2015; Layard et al. 2013), chronic disease, and death (Rosella et al. 2019). Therefore, it is crucial to understand how social milestones in the context of romantic relationships (i.e., transitions including entering a relationship, moving in together, getting married) can be related to life satisfaction trajectories. Although the traditional path in adult romantic life—from singlehood to marriage—has long been considered the norm in Western cultures, this sequence is increasingly challenged by the rise of pre‐marital cohabitation in the past decades (Perelli‐Harris and Bernardi 2015). Changing social norms related to life transitions provide culturally determined sequences that determine not only what events to expect, but also when and in what order they occur. In most cases, entering a relationship with a new partner occurs before moving in together, while marriage can—but does not necessarily have to—take place much later after several years compared to the prior transition.

Stack and Eshleman's (1998) pioneering cross‐national study across 17 countries revealed that adult singles may generally report lower life satisfaction than their married counterparts. Moreover, there is evidence that people living alone are more likely to be affected by a range of mental health problems when compared with their counterparts who live in a shared household (McElroy et al. 2023). This suggests that singles living alone are a high‐risk group for poor mental health, but also that—traditionally seen—marriage is considered to be associated with higher life satisfaction, even if it is only for a short time, as this increase may only last for a few years before returning to pre‐marriage levels (Grover and Helliwell 2019; Lucas and Clark 2006; Luhmann et al. 2012; Qari 2014; Tao 2019).

## 1 Personality Traits and Life Satisfaction in Singlehood

Research on lifelong singles has shown that individuals who have never been in a serious romantic relationship tend to report lower levels of life satisfaction than those who are or have been married (Stahnke and Cooley 2021). Retrospective life course studies further suggest that individuals who have experienced long‐term marriages indicate slightly higher life satisfaction in later life than those who have remained single or faced repeated relationship instability, while the latter two groups do not differ significantly from one another in their reported well‐being (Purol et al. 2021). Stern et al. (2024) show that never‐partnered singles score lower not only in life satisfaction but also in extraversion, conscientiousness, and openness compared to ever‐partnered individuals later in life. Notably, the effect for openness is specific to those who had never been in any serious romantic relationship and did not extend to other operationalizations of lifelong singlehood, such as never having cohabited or never having married. Bühler et al. (2024) report that entering a new partnership is moderately positively associated with life satisfaction—and, to a lesser extent, with conscientiousness—while marriage is associated with a smaller increase in life satisfaction and a decrease in openness at the same time. Together, these findings suggest that stable between‐subject differences may help explain why some individuals remain single across the lifespan—and why this life path is associated with slightly lower life satisfaction on average, possibly not merely as a consequence of lacking a partner, but as part of broader, trait‐like profiles. However, relationship status has also been shown to account for additional variance in life satisfaction beyond personality traits such as neuroticism, suggesting that transitions in the context of romantic relationships may also have a unique association with life satisfaction (Hoan and MacDonald 2024). Uunk and Hoffmann (2023) also report mixed findings: while personality traits did not moderate the positive effects of entering a new partnership, higher levels of neuroticism amplified the negative effects of separation.

After all, there is a consensus that significant interindividual differences not only exist but may reflect a dynamic interplay between personality traits and the social environment (Chopik et al. 2023; Neyer et al. 2014; Stern et al. 2024). Given evidence that life events can trigger at least short‐lived boosts in life satisfaction (e.g., Luhmann et al. 2012; Lucas and Clark 2006), an open question is how large—and how enduring—these fluctuations are when individuals move from one transition to the next within the process of romantic relationship development. Accordingly, Krämer et al. (2025) argue that events occurring in the same context typically unfold in close succession and are conceptually interlinked, such that they should be analyzed jointly rather than in isolation, while emphasizing the importance of accounting for effects due to prior relationship events (e.g., considering the effects on life satisfaction due to the start of a new relationship when analyzing the effects of cohabitation).

## 2 Following the Typical Romantic Path: Which Events Contribute to Life Satisfaction and Which Don't

Increases in life satisfaction are reliably associated with marriage, but growing evidence suggests that much of this potential boost could stem from the anticipation of forthcoming transitions and hence start already with prior events (e.g., Clark et al. 2008; Luhmann et al. 2012; Krämer et al. 2025). Nevertheless, the precise magnitude of these changes remains contested once the full trajectory—entering a partnership, cohabitation, marriage—is taken into account and standardized between individuals. Some studies have pointed to marriage as the key driver of life satisfaction improvements (e.g., Lucas and Clark 2006), while others suggest that prior transitions during the time of entering a new romantic partnership already account for the largest gains (Bühler et al. 2024). Furthermore, evidence from long‐term longitudinal studies shows that cohabitation and marriage may yield comparable increases in life satisfaction when relationship quality and selection effects are accounted for in Western societies (Perelli‐Harris et al. 2019; Næss et al. 2015). Krämer et al. (2025) found that entering a new partnership was associated with life satisfaction increases lasting more than 3 years. In the case of cohabitation, life satisfaction gains emerged in the year following the transition and persisted, suggesting a long‐lasting association. In contrast, the effect of marriage peaked in the year after the event and subsequently declined; though it remained significantly above pre‐event levels. This prompts consideration of whether marriage is uniquely associated with higher life satisfaction beyond the improvements linked to earlier transitions.

## 3 The Present Study

In this study, we examine the associations between entering a relationship with a new partner and moving in together—with and without marriage—and life satisfaction over a 35‐year period using data from large, nationally representative panel surveys from Germany and the United Kingdom.

First, there is a need for studies that disentangle the links between key events in the context of romantic relationships and life satisfaction through direct comparison with each other, while modeling the entire typical sequence from being single and living alone before entering a new romantic relationship to cohabitation and eventually marriage, using a standardized event‐based timeline to enable comparisons across individuals. Although previous research has offered valuable insights and emphasized the importance of accounting for prior relationship events (Krämer et al. 2025), we aim to specifically focus on singles, especially during times when they were living alone before they engage in a romantic relationship. Furthermore, by using nationally representative panel data from Germany and the UK, our study enables cross‐national comparisons between two major Western societies and thus a more comprehensive understanding of these transitions in these contexts. Second, only limited attention has been given to how the effects of cohabitation and subsequent marriage on life satisfaction may have evolved over recent decades in Western societies. This raises the question of whether getting married after moving in with a partner may have been differently associated with life satisfaction in earlier decades when social norms about cohabitation without marriage were different (McIntyre 2014) or factors like the Internet played little to no role in partner selection, compared to more recent years (Rosenfeld 2018). Third, existing research has examined gender differences in the impact of relationship events such as marriage on life satisfaction, yielding mixed results. For instance, while earlier studies suggested that men gain greater psychological benefits from marriage, particularly in terms of well‐being (Gove et al. 1983; Bernard 1982), more recent longitudinal research indicates that women may experience greater benefits (Stutzer and Frey 2006; Tao 2019). Conversely, the negative relationship between singlehood and life satisfaction appears to be more pronounced in men than in women, as well as in younger people than in older people (Stern et al. 2024). There is also evidence of age differences, with individuals marrying at an age below 40 years benefiting more in terms of their psychological well‐being than individuals who marry later in life (Stutzer and Frey 2006). Furthermore, there might be other factors such as income and level of education playing a pivotal role in affecting people's level of life satisfaction regardless of or in addition to their relationship status (Fernández‐Ballesteros et al. 2001; Stern et al. 2024). To build on these prior insights and explore remaining questions, our study has the following three objectives:

*First*, we investigate whether life satisfaction is associated with increases during the full transition sequence from being single living alone to entering a new relationship and living together with a partner, while accounting for the prior co‐occurring events by directly testing them as moderators (specifically, (1) interaction between entering a relationship and the time points around the event of moving in together, and (2) interaction between the time points around the event of moving in together and marriage on life satisfaction) and to what extent this increase is maintained during the first 3 years of cohabitation (*Research Question 1*).

*Second*, we exploratively investigate to what extent the less probable, but still possible, case of an additional marriage could explain an additional proportion of the change in life satisfaction beyond that explained by finding a partner and moving in together (*Research Question 2*). We specifically investigate how the time period between 1984 and 2019 moderates the association between these co‐occurring events and life satisfaction; hence, also capturing trends over time (McIntyre 2014; Rosenfeld 2018).

*Third*, we examine how potential changes in life satisfaction after moving in together vary according to individual's age, gender, socioeconomic status (i.e., different levels of education and net income), and country of residence (UK and Germany; *Research Question 3*).

## 4 Method

### 4.1 Data

The data used in this study come from two large‐scale nationally representative longitudinal panel studies from Germany and the United Kingdom, with annual assessments: The German Socio‐Economic Panel (SOEP; wave 1–35; years of data collection: 1984–2019, *N* = 99,131; Goebel et al. 2019; Brücker et al. 2017; Brücker et al. 2014) and the UK Household Longitudinal Study (UKHLS; wave 1–10; years of data collection: 2009–2019, *N* = 87,045; University of Essex, Institute for Social and Economic Research 2021). To ensure consistent measurement of singlehood status, we excluded the first two waves (years of data collection: 2009, 2010) of the UKHLS data set, as key variables needed to determine romantic relationship status outside the household were not yet included in these early waves. The two surveys collect a wide range of information on topics including income, employment, and well‐being. Because of the potential impact of the COVID‐19 pandemic both on well‐being (e.g., O'Connor et al. 2021) and cohabitation patterns (e.g., Langenkamp et al. 2022) we use data up to and including 2019.

### 4.2 Sampling Strategy

**FIGURE 1:**
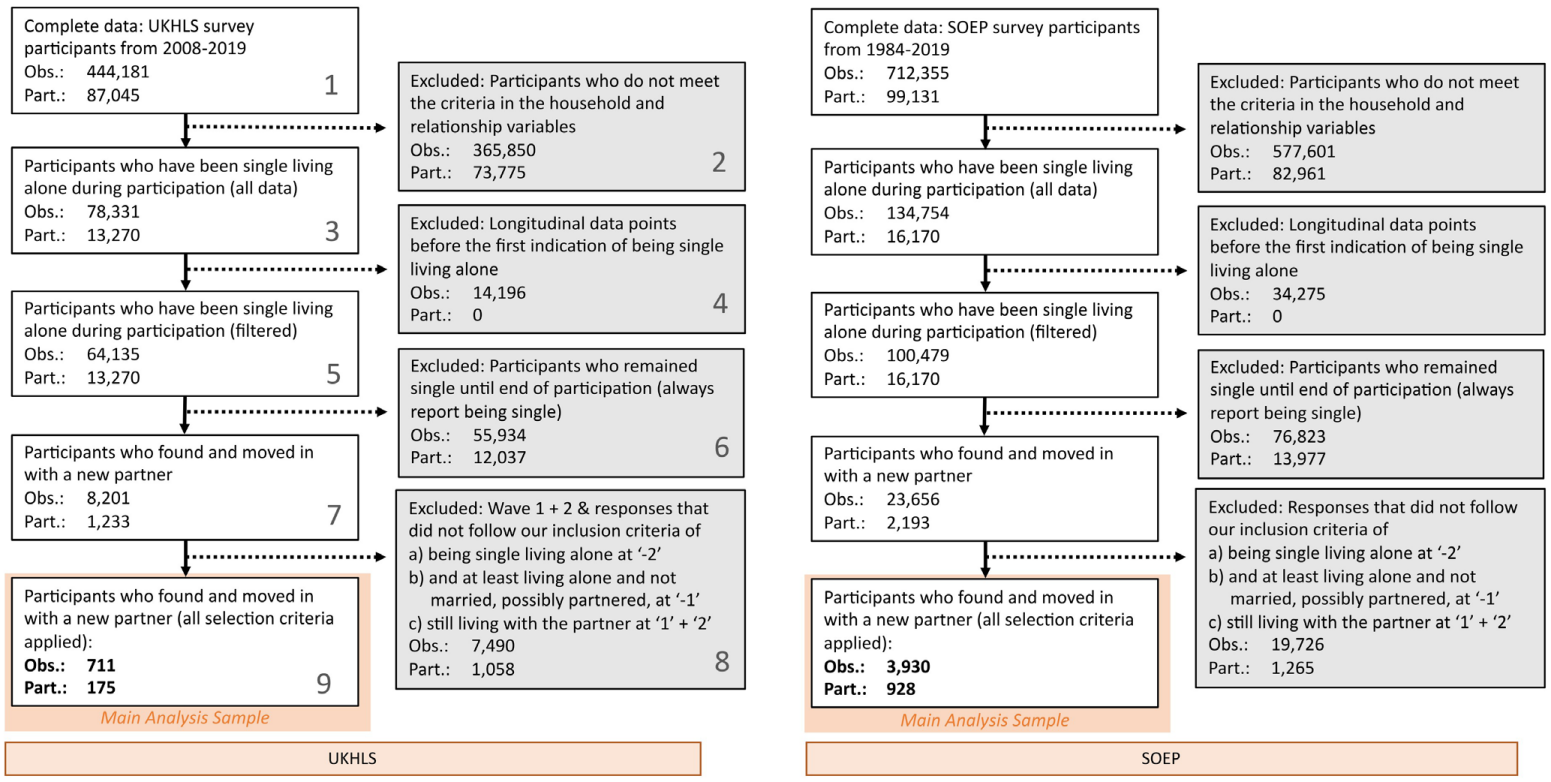
Participant flow charts for the UKHLS (left) and SOEP (right) samples. Each chart shows the number of observations (Obs.) and unique participants (Part.) retained or excluded at each step of the selection process. The final sample includes individuals who (a) were single and living alone at time point “−2”, that is, approximately 2 years after moving in together, (b) were still living alone and not married at time point “−1”, that is, approximately 1 year before moving in together, even if a romantic relationship may have already existed, and (c) were living with a new partner at both time points “1” and “2”, that is, approximately 1 and 2 years after moving in together (see Step 8).

The target group for this study comprised those adult participants (at least 18 years old) from the total sample consisting of the merged UKHLS and SOEP data who had indicated that they lived alone and were not in a romantic relationship at any wave during their participation in the study (see Figure 1, Step 3). Only participants who fulfilled this constellation at least once were considered from the wave on which this has been first reported (see Figure 1, Step 5). Data from earlier waves were excluded to ensure a uniform baseline condition across individuals. Variables that provide information on the number and specific relationship to persons living in the household, or directly on household composition, such as “man, 65+ years, no children” or “living as a couple” (UKHLS), were used to infer household situation. Both data sets have variables indicating the relationship status (marital and non‐marital), such as single, married, or divorced, at each wave. Moreover, both data sets also captured information on non‐marital romantic relationships with partners living outside the household (this information was available in the UKHLS starting in Wave 3). This allowed us to more precisely identify genuinely singles by excluding those who were formally unpartnered but nonetheless in a steady relationship with someone they did not live with (e.g., “Are you in a serious/permanent relationship?” and “Partner lives in household” in SOEP; “Do you have a steady relationship with someone you are not living with here, whom you think of as your ‘partner’” in UKHLS).

In order to ensure an exclusive focus on individuals with a typical sequence (starting with being single while living alone, finding a partner, moving in together, and eventually marriage), only participants who later reported entering a romantic relationship and moving in with a new partner—without children in the household (as living with children, e.g., from a previous relationship, could independently affect life satisfaction, see Bechetti et al. 2013)—were included in the study (see Figure 1, Step 7). In addition, they had to meet the criteria of (a) being single living alone at least two waves prior to moving in together and (b) living alone at one wave prior to moving in together, despite possibly (but not necessarily) being in a romantic relationship already (see Figure 1, Step 8). We excluded individuals who had already married before moving in with their new partner (*n* = 24). Finally, (c) we then excluded observations of individuals who, in the next 2 years after moving in with their partner, returned to singlehood or living alone, moved in with others such as children or new partners, or otherwise deviated from the cohabiting partnership structure. Importantly, we excluded participants with intermittent participation between 2 years before and 2 years after the cohabitation transition, as such gaps would have introduced ambiguity regarding their relationship or household status during missing waves. Table 1 shows the descriptive statistics including demographic information on participants in the analysis sample.

**TABLE 1:** Demographic information on participants in the analysis sample.

	UKHLS sample	SOEP sample	Pooled sample
*N*_total_	175	928	1103
Obs._total_	957	10,367	11,324
Obs._valid (“–2” to “2”)_	711	3930	4641
Age_total_, M (SD)	41.80 (13.61)	37.7 (13.87)	38.35 (13.87)
Age_under 40_, *N*_total_ (%)	104 (59.4)	657 (70.8)	761 (69.0)
Age_40–59_, *N*_total_ (%)	54 (30.9)	194 (20.9)	248 (22.5)
Age_over 60_, *N*_total_ (%)	17 (9.7)	77 (8.3)	94 (8.5)
Gender_women_, *N*_total_ (%)	81 (46.3)	402 (43.3)	483 (43.8)
Educational attainment, *N*_total_ (%)			
Less than H S	6 (3.7)	103 (11.4)	109 (10.1)
H S Level	53 (32.9)	584 (63.8)	637 (59.3)
More than H S	102 (63.4)	227 (24.8)	329 (30.6)
Net income, *N*_total_ (%)	158 (90.4)	855 (92.1)	1013 (91.8)
Net income, M_total_ (SD)	1740 £ (924 £)	2118 € (1634 €)	—
LS stand., M_total_ (SD)	0.02 (0.99)	−0.01 (1.0)	0 (1.0)

*Note:* Obs._total_ = all observations from study participants who met the inclusion criteria across their full period of participation. Obs._valid (“−2” to “2”)_ = all data points falling within the “–2” to “+2” time frame. H S = high school. Educational attainment as measured in SOEP; for UKHLS: Less than H S = participants without qualification, H S Level = participants with A‐Level, GCSE, and other qualifications, More than H S = participants with degree and other higher degrees. Income as measured in UKHLS (net income); for SOEP as measured by individual labor income. LS (stand.) = life satisfaction (*Z‐*scores) as standardized in the pooled sample.

### 4.3 Measures

A comprehensive list of all variables, including response scales and harmonization across survey waves and datasets (SOEP, UKHLS), is provided in the Data S1.

### 4.4 Relationship Status and Household Composition

To track relationship status over time, longitudinal data from both UKHLS and SOEP surveys was used. Of particular interest were variables indicating single status (e.g., “single,” “separated,” “divorced,” “widowed”) or the existence of a marital and non‐marital romantic relationship (e.g., “living as a couple,” “married,” “same‐sex civil partnership,” “in a serious/permanent relationship,” “a steady relationship with someone”). This data was combined with information from variables concerning the household situation, if such information was not already available. Variables indicating whether participants were living with a spouse or an unmarried partner—i.e., someone they considered their romantic partner—were also taken into account (see Section 4.2). Additionally, variables indicating the number of individuals and children in the household were examined. Taken together, this information was used to create a harmonized variable for each year, categorizing participants as follows: (a) living alone and not in a romantic relationship, (b) living alone but in a romantic relationship, (c) living with their partner, or (d) living with their partner and children. Participants in category (d) were excluded from the analysis.

### 4.5 Life Satisfaction

Life satisfaction was measured annually with the item “How satisfied are you with your life in general?” Participants were asked to respond on a 7‐point scale in UKHLS with anchors at 1 (*Completely dissatisfied*) and 7 (*Completely satisfied*), and on an 11‐point scale in SOEP ranging from 0 (*Completely dissatisfied*) to 10 (*Completely satisfied*). *Z*‐scores were calculated from the raw values for each data set as well as after merging the data.

### 4.6 Potential Confounders and Moderators

Potential moderators encompassed socio‐demographic factors: age was included as a continuous *z*‐standardized variable in the models. For the purpose of visual representation, however, it was categorized into three groups: young adults (39 years and younger), middle‐aged adults (40–59 years), and older adults (60 years and older). Gender was classified as woman or man (= reference). Monthly net income at the individual level (including primary and secondary employment as well as self‐employment) was included as a continuous *z*‐standardized variable in the models and categorized into three groups (“low,” “medium,” and “high”) based on tertiles for the purpose of visual representation. Educational attainment was harmonized across the two data sets to ensure comparability. Specifically, UKHLS categories such as “Degree” and “Other higher degree” were aligned with the SOEP category “More than High School”; “GCSE” and “A‐level” were matched with “High School”; and “No qualification” was categorized as “Less than High School.” These three levels formed the final classification used in the analysis.

### 4.7 Analytical Strategy

This study adheres to the APA Journal Article Reporting Standards for Quantitative Research (JARS‐Quant; Appelbaum et al. 2018) to ensure transparency in research design, data collection, and analysis. All analyses were carried out with R version 4.3.1 (R Core Team 2022) and mainly with the packages nlme (Pinheiro et al. 2023) and lme4 (Bates et al. 2015). For all analyses, a significance level of α = 0.05 was adopted, with results considered statistically significant at *p* < 0.05. The analysis script used in this study is publicly available on OSF (EL‐Awad and Eves 2025; https://osf.io/438t6.). A series of linear mixed‐effects models (LMM) were then calculated using the merged data, with life satisfaction as the outcome variable (Table S1 presents an overview of the analysis models, including their formulas using Wilkinson notation). The main predictor was a categorical variable with five values “−2” to “2,” where “0” represents the measurement time (or year) when singles who were previously living alone reported having moved in with a new partner for the first time, “−2” and “−1” respectively indicate 2 and 1 years before moving in together, “1” and “2” for 1 and 2 years after moving in together, respectively.

#### 4.7.1 Analysis Related to Research Question 1

To account for within‐person variability, the models included random intercepts at the participant level. Random slopes were omitted to ensure model stability and interpretability, following recommendations for designs with a limited number of repeated measures per cluster (Matuschek et al. 2017). Marginal means were contrasted, and standardized mean differences (expressed in SD units) were used as effect sizes to facilitate the interpretation of life satisfaction changes across time points. To control for multiple comparisons, *p*‐values were adjusted using the False Discovery Rate (FDR) procedure according to Benjamini and Hochberg (1995), particularly for repeated contrasts across the five time points. We additionally tested robustness through a stepwise model‐building approach, in which key socio‐demographic covariates (i.e., gender, age, net income, education, and country of residence) were sequentially included in the fully adjusted model. Model comparisons were performed by comparing AIC and BIC values to identify time points that were more sensitive to covariate inclusion, especially income and education. To further examine the robustness of findings related to changes in life satisfaction across time points, an additional model only including within‐person *z*‐standardization of life satisfaction scores as an outcome was applied as a sensitivity analysis. This approach isolated within‐individual fluctuations over time while controlling for stable between‐person differences. Finally, to investigate whether moving in together contributes to life satisfaction beyond the effect of entering a relationship, we conducted an additional analysis focusing exclusively on the subgroup of individuals who had already found a partner 1 year before moving in together (*N* = 573; M_age_ = 38.46, SD_age_ = 13.56; 44.3% women). We tested whether life satisfaction increased significantly from the time of entering a romantic relationship (at “−1”) to moving in with a new partner (at “0”) and compared both phases with the period of being single and living alone (at “−2”). In addition, we analyzed and visualized the interaction between entering a relationship and moving in together on life satisfaction among former singles living alone.

#### 4.7.2 Analysis Related to Research Question 2

We aimed to examine whether, and to what extent, the association between moving in with a partner and life satisfaction was affected by getting married at the same time or shortly thereafter by analyzing potential interaction effects between the events. With this, we sought to determine whether marriage provides an additional positive impact on life satisfaction beyond cohabitation. A binary predictor was created to indicate whether a participant got married when or shortly after moving in with a new partner within time points “0,” “1,” and “2” (but not at time point “−1”). Estimated marginal means of life satisfaction were then calculated and contrasted to the two time points prior to moving in with the new partner (at “−2” and “−1”). In addition, a sensitivity analysis was conducted comparing participants who married in the same year as cohabitation (at “0”) with those who did not marry during the observation window. This analysis tested whether the temporal proximity of marriage to cohabitation had an effect on well‐being trajectories. Since the SOEP survey provides a uniquely long observation period of 35 years, we conducted additional analyses using this data set to test the interaction between marital status, time points relative to moving in with a new partner, and historical period effects (i.e., the exact year of cohabitation), allowing us to capture potential societal shifts in the marriage‐life satisfaction link.

#### 4.7.3 Analysis Related to Research Question 3

To test whether potential changes in life satisfaction during the time around the event of moving in together were associated with socio‐demographic factors, interaction terms with age, gender, monthly net income, the educational attainment level, and the country of residence were included in the LMMs; marginal means were contrasted. To evaluate the stability of detected moderators, we ran a series of robustness analyses, in which each sociodemographic variable was interacted with the time variable while other covariates were removed step by step. The two significant moderators—age at time point “–2” and income at “–1”—were consistently replicated across these models. In this context, missing values were predominantly present in the socio‐economic variables of net income and educational attainment data, ranging on average between 1% and 2% of the data from the target groups relevant for the analyses. Given the minor proportion of missing values, the large sample sizes, and the reduced likelihood of biased outcomes as a result, we chose to conduct all models using the fully available data.

## 5 Results

### 5.1 Participants

In total, we identified *N* = 27,459 individuals who reported being single and living alone at least once. Based on the sampling strategy as described in the Method section (see also Figure 1), a final sample size for participants who were initially single living alone, entered a romantic relationship with a new partner, and moved in together was *N* = 1103 (M_age_ = 38.35 years, SD_age_ = 13.87, 43.8% women). See Table 1 for more information on the final sample.

#### 5.1.1 Research Question 1

*Changes in life satisfaction during and after the transition from being single and living alone to entering a relationship and cohabitation*. Life satisfaction was highest at “0,” in the year participants moved in with their new partner (see Plot A in Figure 3). In that year, life satisfaction scores were 0.35 SD higher (95% CI [0.29–0.41], *p* < 0.001) than in the 2 years prior to cohabitation at “−2,” and 0.16 SD higher (95% CI [0.10–0.22], *p* < 0.001) than 1 year before at “−1”. Compared to the peak at “0,” life satisfaction showed a slight decline in the following years. This was statistically significant 2 years after the transition (−0.09 SD, 95% CI [−0.14 to −0.01], *p* = 0.021), but not 1 year after (−0.07 SD, *p* = 0.052; FDR‐adjusted).

Furthermore, Table S3 presents the model comparisons with stepwise inclusion of covariates as a robustness check. At time point “1,” the effect becomes non‐significant when age is added (−0.06 SD, not significant), accompanied by higher AIC and BIC values. At time point “2,” the effect turns non‐significant with the inclusion of income and education. AIC and BIC improve notably with these covariates but increase again when country is added. Estimates at time points “–2” (≈−0.35 SD) and “–1” (≈−0.16 SD) remain consistently significant across all specifications (all *p*s < 0.001). The sensitivity model using within‐person standardized life satisfaction scores confirmed the pattern: life satisfaction peaked in the year of moving in together (timepoint “0”) and was significantly higher than in all other years (all *p*s < 0.001, FDR‐adjusted). Specifically, life satisfaction at “0” was 0.48 SD above the reference at “–2” (95% CI [0.41–0.55]), 0.21 SD above “–1” (95% CI [0.14–0.28]), 0.12 SD above “1” (95% CI [0.04–0.20]), and 0.17 SD above “2” (95% CI [0.09–0.26]). No statistically significant differences were found between the 2 years following moving in together (i.e., “1” vs. “2”: 0.05 SD, *p* = 0.261).

**FIGURE 2:**
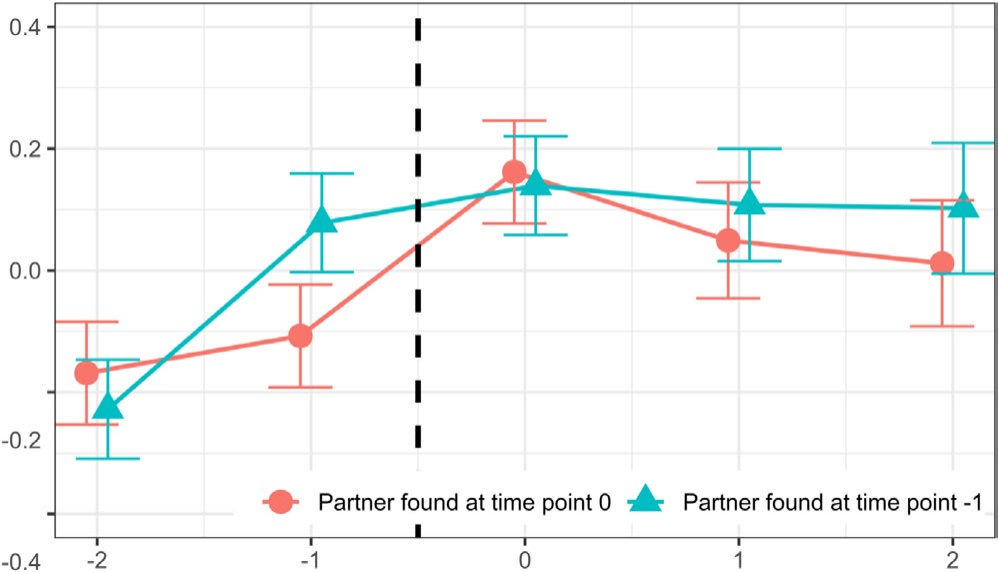
*Y*‐axis: estimated means of life satisfaction, *x*‐axis: time point of moving in with a new partner (at “0”), approximately 2 and 1 years before the event (at “–2” and “–1”), and approximately 1 and 2 years after the event (at “1” and “2”), showing estimated means relying on mean *Z*‐scores.

In addition, the post hoc analysis with a subset of participants who had already found a partner at “–1” revealed that, in this case, no significant changes in life satisfaction occurred between the year of moving in together at “0” and the adjacent years. Life satisfaction was significantly higher in the year of cohabitation compared to “−2” (0.37 SD, 95% CI [0.28–0.45], *p* < 0.001), but no significant differences were found relative to “–1,” “1,” or “2” (all *p*s > 0.26, FDR‐adjusted). In line with these results, the interaction plot (see Figure 2) shows a significant difference (0.19 SD, 95% CI [0.06‐0.30], *p* < 0.001) in life satisfaction at time point “–1” between participants who had already found a partner and those who had not. Specifically, individuals who were already in a relationship prior to the year of moving in together reported substantially higher life satisfaction 1 year before cohabitation. Furthermore, the increase in life satisfaction among those who had already found a partner before time point “0” remained the same 2 years later at “2”, as there was no significant decline in this group—in contrast to those who had not yet found a partner, who reported a significant decline of −0.15 SD (95% CI [−0.30 to –0.05], *p* = 0.009).

#### 5.1.2 Research Question 2

**FIGURE 3:**
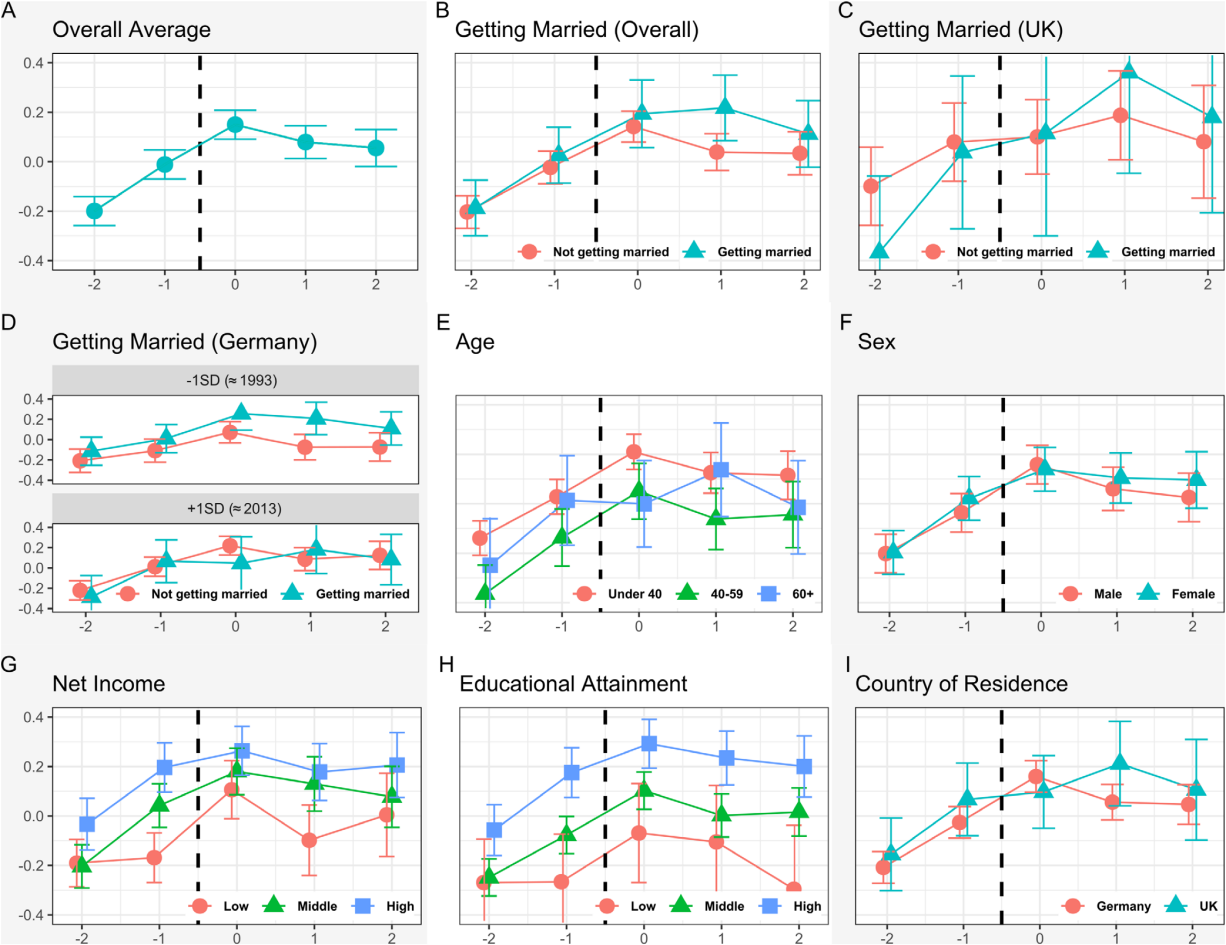
*Y*‐axis: estimated means of life satisfaction, *x*‐axis: time point of moving in with a new partner (at “0”), approximately 2 and 1 years before the event (at “–2” and “–1”), and approximately 1 and 2 years after the event (at “1” and “2”). All plots showing estimated means relying on mean *Z*‐scores. Plot B/C/D: Not getting married/Getting married = participant did not marry/married at time points “0,” “1,” or “2”. Plot E: “Under 40”, “40‐59” and “60+” refer to to participants’ age groups in years (< 40, 40–59, ≥ 60). Plot D: “−1 SD” and “+1 SD” refer to one standard deviation below and above the mean year of the event of moving in together with a new partner across the sample (Mean ≈ 2003), corresponding approximately to 1993 and 2013, respectively. Plot G: Net income low/middle/high cut‐offs by tertiles. Plot H: Educational attainment low/middle/high = Less than High School/High School/More than High School. Plot I: Germany = SOEP data; UK = UKHLS data.

*Examining the extent to which marriage, as a common co‐occurring event, explains additional changes in life satisfaction beyond those attributable to finding a partner and moving in together*. The analyses revealed no consistent or statistically significant interaction effects between any time points around the event of moving in together and marital status on life satisfaction, while controlling for age, gender, net income, educational attainment, and country of residence (see Table S5). Results remained virtually unchanged when control variables were removed one by one, indicating that the absence of a marital effect is robust across model specifications. However, contrast analyses of estimated marginal means showed that participants who married reported significantly higher life satisfaction at time point “1” (1 year after moving in) compared to those who did not marry (difference = 0.23 SD, 95% CI [0.10–0.50], *p* = 0.005; see Plot B in Figure 3). No significant differences between married and non‐married participants were observed at the other time points (−2, −1, 0, and 2; all *ps* > 0.12). In SOEP data, a significant difference in life satisfaction between those who married and those who did not (difference = 0.30 SD, 95% CI [0.04–0.49], *p* = 0.018) was observed only during the earlier period (one standard deviation below the mean of the study period, approximately 1993), but not in the more recent period (one standard deviation above the study duration mean, approximately 2013; see Plot D in Figure 3 and Table S8). In the years prior to moving in together, at “−2” and “−1”, no significant differences were found in the estimated marginal mean *z*‐scores of life satisfaction between participants who married and those who did not marry during the study period (see Plot B—D in Figure 3 and Table S5). A stratified sensitivity analysis was conducted exclusively with participants who married in the same year in which they moved in together versus participants who did not marry during the study period also revealed no significant differences (see Table S6).

#### 5.1.3 Research Question 3

*Changes in life satisfaction before and after moving in with a partner in different socio‐demographic groups*. The analysis of socio‐demographic factors (i.e., age, gender, education attainment level, net income, and country of residence) revealed very few significant interactions between the relative timing of moving in together and these factors (see also Plots E–I in Figure 3; Table S4 for the fully adjusted model with merged data; Table S7 for the UKHLS data analysis and Table S8 for the SOEP data analysis in the Data S1). Age was a significant moderator at “−2,” with older participants reporting lower life satisfaction than younger participants (−0.15 SD, 95% CI [−0.25 to −0.05], *p* = 0.002). Furthermore, participants with a net income one SD above the mean reported on average 0.14 SD higher life satisfaction at “−1” (95% CI [0.06–0.22], *p* < 0.001). This is also shown in the visualization of life satisfaction patterns based on the categorization of net income groups (see Plot G in Figure 3). The robustness check in which each socio‐demographic factor was interacted with the time points around “0”, while sequentially removing other covariates confirmed that the two previously identified significant moderation effects—age at “−2” and income at “−1”—were replicated across all model specifications. In contrast, factors that had shown no significant interactions in the fully adjusted model (e.g., gender, education, country) remained consistently non‐significant across all robustness analyses.

## 6 Discussion

Singles in Germany and the UK who moved in with a new partner reported a significant increase in life satisfaction in the year of cohabitation, with a standardized gain of up to 0.48 SD. This effect was statistically robust and replicated across models, including a within‐person specification that controls for stable between‐person differences. This suggests that cohabitation is associated with a particularly salient moment in the romantic trajectory. However, the increase in life satisfaction did not continue at the same rate in all subsequent years. Although life satisfaction remained above the level prior to cohabitation, the effect strength declined slightly, and some comparisons (e.g., between time points “0” and “1” or “2”) did not consistently achieve statistical significance across all model specifications. The fact that some effects become non‐significant when demographic covariates are added does not suggest that they vanish entirely, but rather that part of the variance is absorbed by between‐person characteristics. This underlines the importance of within‐person modeling, which adjusts for stable traits and supports the robustness of the peak in life satisfaction around the transition to cohabitation. Therefore, the effect appears to persist in the medium term, but not clearly over a three‐year period.

Nevertheless, our findings on the extent of life satisfaction increases after moving in with a new partner are somewhat in line with research on the “honeymoon phase” often shown based on the event of marriage, during which life satisfaction temporarily peaks (e.g., Lorber et al. 2015). This increase is generally attributed to emotional responses, fulfillment of psychological needs, and changes in social status due to relationship progression (Acevedo and Aron 2009; Mutz and Kämpfer 2013). However, we found no evidence for a decline in life satisfaction during the years following cohabitation—despite theoretical expectations that such decreases might occur due to a recalibration of expectations or a shift from an idealized to a more realistic perception of the relationship (e.g., Neff and Buck 2023). In contrast to prior studies grounded in set‐point theory, which suggest that life satisfaction typically returns to baseline within 2 years of major romantic transitions (Asselmann and Specht 2023; Clark et al. 2008; Lucas and Clark 2006), our findings suggest a sustained increase in well‐being—at least in the medium term—following the formation of a new romantic relationship and subsequent cohabitation. This pattern was particularly evident among individuals who had already entered a relationship prior to moving in together and remained in this stage for a considerable time before experiencing further transitions.

Importantly, entering a relationship earlier may already be associated with higher psychological well‐being, such as increased life satisfaction due to emotional stability, social support and a positive outlook for the future. Indeed, participants who had already found a partner 1 year prior to cohabiting (at “−1”) reported significantly higher life satisfaction than those who had not yet entered a relationship, with an estimated difference of 0.19 SD. For these individuals, life satisfaction was already elevated prior to moving in together and remained stable afterwards. Thus, rather than cohabitation causing a further rise, it appears that the major increase in well‐being occurred during relationship formation, consistent with the view that anticipation and emotional investment precede formal transitions (Clark et al. 2008; Luhmann et al. 2012; Krämer et al. 2025). This may be especially true for our sample, which was restricted to individuals undergoing relationship formation and cohabitation in close succession—likely reflecting partnerships characterized by greater stability and stronger anticipation effects. Taken together, these findings indicate that entering a romantic relationship represents the primary psychological turning point for former singles who were living alone, while cohabitation may be linked to the maintenance or even enhancement of prior well‐being gains—particularly when a longer period elapses between relationship formation and moving in together, allowing expectations and emotional investment to build.

Overall, marriage in the first year of cohabitation or shortly thereafter had only a small and temporary positive effect on life satisfaction—and this was only in a specific part of the sample. Specifically, a significant difference was only found in the earlier survey waves of the SOEP data set, around 1993. In later survey periods and in the UKHLS data, this effect was no longer detectable, which could indicate a cultural and historical change in the importance of marriage for subjective well‐being. Marriage may no longer be experienced as a central symbol of commitment today, as unmarried cohabitation is increasingly socially accepted (Sassler and Lichter 2020). In the fully adjusted models, there were no consistent differences in life satisfaction between participants who married in connection with moving in together and those who did not. This suggests that marriage has limited additional benefits in the context of existing partnerships. Analysis of the period prior to cohabitation also revealed no systematic differences in well‐being between couples who later married and those who remained unmarried. This suggests that both groups were in comparable life situations before marriage and did not differ significantly in terms of their subjective well‐being. The declining importance of marriage for individual well‐being is consistent with Cherlin's (2004) theory of the deinstitutionalization of marriage, according to which traditional norms and expectations surrounding marriage are becoming increasingly less binding. This development has since been observed in other countries (Treas et al. 2014) and reflects a cultural shift in which many couples find cohabitation without marriage just as fulfilling and stable as traditional marriage.

In general, patterns of change in life satisfaction were similar across most sociodemographic groups. Notably, no significant gender differences were found in life satisfaction trajectories before and after moving in with a partner. However, there were some notable exceptions to this general trend. In the year before moving in together, individuals with higher net income reported higher life satisfaction than their counterparts with lower income (difference = 0.14 SD). Additionally, individuals with lower income experienced a sharper decline in life satisfaction in the years following the initial increase. This finding is consistent with prior research showing that financial stress has been linked to lower well‐being, particularly among economically vulnerable individuals (Brzozowski and Spotton Visano 2020). Interestingly, no clear educational differences in life satisfaction emerged in the fully adjusted models. While prior research has shown mixed findings on this topic, our results suggest that education may play a smaller or more context‐dependent role in explaining life satisfaction changes around relationship transitions. In Germany, on the other hand, age played an important role for singles living alone who were to move in with a new partner 2 years later: older participants reported lower life satisfaction than younger ones, suggesting a possible moderating effect of demographic factors given different life stages. This moderation effect was specific to the pre‐transition phase and did not extend to the years after cohabitation. However, research by Park et al. (2022) found that satisfaction with singlehood tends to increase after midlife rather than decline linearly with age, indicating that age‐related effects on well‐being among singles may be more nuanced and dependent on life stage and societal contextual factors at the societal level (Realo and Dobewall 2011). The singles in our study differed in that they were on the verge of entering a new relationship and moving in with a partner.

## 7 Strengths and Limitations

Our study has several notable strengths. We combined long‐term and representative household survey data from Germany and the UK, covering 35 years, which enabled us to closely examine changes across lifespan and different time periods. Unlike most previous studies that analyzed the effects of co‐occurring events—such as finding a new partner, moving in together, and getting married—on life satisfaction separately, we were able to disentangle the effects of these events by following a standardized transitional design ensuring internal validity and comparability.

However, we also must recognize some important limitations. First, while single‐item measures are often considered more susceptible to measurement error, context effects, and mood influences, research has shown that they demonstrate comparable reliability and validity to multi‐item scales (e.g., Cheung and Lucas 2014; Realo and Dobewall 2011). Second, although the strong selection through the application of strict inclusion criteria strengthens internal validity and makes causal inferences more robust, it comes at the expense of external validity, making generalization significantly more difficult. Third, our findings are based on correlational data, and that does not suffice to draw causal conclusions. We also point out that the strict sample filtering could have reduced the generalizability of the findings. Moreover, we have clarified that potential causal interpretations would require additional assumptions that we do not claim to test directly, such as the absence of time‐varying unobserved confounders. Finally, the data for our study come from two highly developed and wealthy, so‐called WEIRD (Henrich et al. 2010) European countries, which limits the generalizability of the results to other cultural and economic contexts.

## 8 Future Research Directions

Future research should investigate the mechanisms responsible for the steeper decline in life satisfaction after moving in together among individuals with lower income compared to those with higher income. Large‐scale cohort studies examining the significance of marriage and cohabitation on life satisfaction could provide valuable information on changes in social norms by comparing different time periods. It has been suggested that individuals with higher levels of extraversion are not only more likely to enter romantic relationships but may also benefit more from being partnered compared to their more introverted counterparts—a notion supported by findings from Chopik et al. (2023) and worth exploring further in future research. Finally, further research across different countries and cultures, especially non‐WEIRD countries, is needed to determine whether similar patterns of change in life satisfaction occur when single individuals find a partner and move in together.

## 9 Conclusion

Starting a romantic relationship and moving in together is associated with a significant rise in life satisfaction, peaking in the year of cohabitation. This gain remains largely stable through the first year post‐move and still lies significantly above the level observed 2 years before cohabitation in the second year, indicating only a modest decline from the peak. These trajectories are consistent across gender, age groups, and between Germany and the UK, although individuals with lower income experience a somewhat steeper drop after the initial boost. Marriage within the same period was associated with a short‐lived additional increase in the SOEP cohort around 1993, but this effect is absent in more recent decades. This shift suggests that, in contemporary Western societies, cohabitation itself may now carry the symbolic commitment once conferred by marriage.

## Author Contributions

U.E.: conceptualization, data curation, formal analysis, writing – original draft. R.E.: data curation, writing – review and editing. J.H.: writing – review and editing. T.M.E.: writing – review and editing. R.G.: writing – review and editing. A.R.: writing – review and editing. S.L.: conceptualization, writing – review and editing.

## Conflicts of Interest

The authors declare no conflicts of interest.

## Supporting information


**Data S1:** jopy70013‐sup‐0001‐DataS1.docx.

## Data Availability

Materials: All study materials are publicly available (https://doi.org/10.5255/UKDA‐SN‐6614‐19 and https://doi.org/10.5684/soep.core.v36eu). Data: All data are publicly available (https://doi.org/10.5255/UKDA‐SN‐6614‐19 and https://doi.org/10.5684/soep.core.v36eu). Analysis script: The analysis script (R‐file) has been uploaded to the Open Science Framework (OSF) and can be accessed or downloaded via the following link: https://osf.io/438t6.
