# Supplementary material for: Mapping Life Satisfaction Over the First Years of Cohabitation Among Former Singles Living Alone in UK and Germany
Source: J Pers. 2025 Aug 18;94(3):446–57. doi: 10.1111/jopy.70013 (PMC13163626; doi:10.1111/jopy.70013)
Supplement: Supplementary file 1 — Data S1: jopy70013‐sup‐0001‐DataS1.docx. [file JOPY-94-446-s001.docx]

**##################################################################################
######## SUPPLEMENTARY MATERIAL ################################################
##################################################################################**

**Mapping life satisfaction over the first years of cohabitation among former singles living alone in UK and Germany**

Usama EL-Awad, Robert Eves, Justin Hachenberger, Theresa M. Entringer, Robin Goodwin, Anu Realo, & Sakari Lemola

**TABLE OF CONTENTS**

**##################################################################################**

1. **Used variables for data preparation**………………………………………………………………………………………1
2. **Used variables for analyses**…………………………………………………………………………………………………..4
3. **Table S1:** Model descriptions and formulas……………………………………………………………………………7
4. **Table S2:** Estimated mean differences in life satisfaction two years before to two years after moving in with a new partner (fixed effects analysis) using merged data (UKHLS & SOEP)…10
5. **Table S3:** Estimated mean differences in life satisfaction two years after moving in with a new partner (fixed effects analysis) using merged data (UKHLS & SOEP) with stepwise inclusion of covariates…………………………………………………………………………………………………………………………10
6. **Table S4:** Estimated mean differences in life satisfaction two years before to two years after moving in with a new partner conditioned by potential sociodemographic moderators (linear mixed model) using merged data (UKHLS & SOEP)……………………………………………………………11
7. **Table S5:** Estimated mean differences in life satisfaction when moving in and marrying vs. not marrying (linear mixed models) using merged data (UKHLS & SOEP)…………………………………13
8. **Table S6:** Sensitivity Analysis: Estimated mean differences in life satisfaction when moving in and marrying in the same year vs. not marrying (linear mixed models) using merged data (UKHLS & SOEP)……………………………………………………………………………………………..…………………14
9. **Table S7:** Estimated mean differences in life satisfaction two years before to two years after moving in with a new partner conditioned by potential sociodemographic moderators (linear mixed model) using UKHLS data…………………………………………………………………………………………..15
10. **Table S8:** Estimated mean differences in life satisfaction two years before to two years after moving in with a new partner conditioned by potential sociodemographic moderators (linear mixed model) using SOEP data………………………………………………………………………………………….17

**##################################################################################**

1. **Used variables for data preparation (see Sampling Strategy in Method)***Note:* Only the content levels are listed, and missing value markers are omitted.
2. **SOEP Variables:**

- **pequiv/d11104**: Marital status.
  This variable indicates the legal marital status of individuals aged 16 and older in the household at the time of the interview. The information is derived from the original SOEP variable YFAMSTD and recoded based on age into a harmonized format. The classification includes legally married individuals as well as those living with a partner (category 1). For married individuals not living with their spouse (e.g., guest workers), values were assigned based on specific age criteria, using codes 6 or 7 in the original variable, which were recoded to 1 in the derived variable D11104. The variable is sourced from the _PGEN file and used for identifying marital status as a covariate in the present analyses.
  1. Married/ Living with a Partner
  2. Single
  3. Widowed
  4. Divorced
  5. Separated
- **pequiv/d11105**: Relationship to household head.
  This variable captures each individual’s relationship to the household head in the respective survey year. It is derived from the original SOEP variable YSTELL and harmonized into five broad categories to ensure consistency across survey years, including waves with more detailed relationship classifications (from wave 29 onward). Prior to wave 29, the variable was generated using a simplified mapping of *YSTELL* codes (e.g., 1 = partner, 3/4 = child). Starting in wave 29, more detailed codes were used to distinguish between different types of relatives and unrelated individuals. In both cases, the derived variable D11105 assigns respondents to one of five relationship groups. The variable is sourced from the _PBRUTTO file and is used in the present study to help determine household structure and identify whether individuals live alone, with a partner, or with other persons.
  1. Head
  2. Partner
  3. Child
  4. Relative
  5. Nonrelative
- **pequiv/d11106**: Number of persons in household.
  This variable indicates the total number of individuals living in the household at the time of the interview. The information is typically provided by the household head or a knowledgeable household member. The variable is derived directly from the original SOEP variable *YHHGR* and is stored in the _HBRUTTO file. It is used in the present study to determine household size, specifically to identify single-person households as part of the sample selection criteria.
- **pequiv/d11107**: Number of children in household.
  This variable indicates the number of individuals under the age of 18 living in the household at the time of the interview (valid values range from 0 to 10, indicating the number of children in the household). It is calculated based on the age variable of each household member and reflects the presence of minors in the household and derived by summing all household members aged 0–17, using the individual-level age variable *d11101* from the $PPFAD file. In cases where age data is missing but the household member was identified as a child (based on *$netto* codes 20–27), they were still counted as minors.
- **pl/pld0133**: Partner lives in household.
  This variable captures whether the respondent’s romantic partner lives in the same household (response scale: yes; no; does not apply) at the time of the interview. It is conditional on the respondent indicating that they are in a serious or permanent romantic relationship (as assessed by variable pld0132_v1 or its harmonized predecessor pld0132_h). If the respondent reports being in such a relationship, they are subsequently asked whether their partner lives with them in the household.
- **pl/pld0137**: Moved in together.
  This variable asks whether the participant moved in together with a partner since January 1st of the calendar year preceding the interview (response scale: yes; does not apply). It is part of a larger question block assessing changes in the respondent's family situation. Respondents are asked to indicate whether specific life changes have occurred (‘Has your family situation changed since January 1, 2021? Please indicate if any of the following have occurred, and if so, when.’).
- **pl/pld0038**: Got together with a new partner.
  This variable records whether the participant has started a new romantic relationship since January 1st of the calendar year preceding the interview (response scale: yes; does not apply). It is part of a larger question block assessing changes in the respondent's family situation. Respondents are asked to indicate whether specific life changes have occurred (‘Has your family situation changed since January 1, 2021? Please indicate if any of the following have occurred, and if so, when.’).
- **ppathl/syear**: Provides information on survey year (e.g., 1984, 1985, …).

1. **UKHLS Variables:**

- **mastat_dv**: De facto marital status.
  The variable mastat_dv represents the de facto marital status of individuals and is a derived variable within the UKHLS dataset. It is created by recoding the original marital status variable (marstat) and incorporating additional information from the variables age_dv, livewith, and ivfio. In doing so, it allows for a more accurate classification of individuals’ relationship status by including two additional groups: (1) children under the age of 16, based on age and household information, and (2) respondents who are not legally married but currently live in a cohabiting partnership.
  1. Child under 16
  2. Single and never married/in civil partnership
  3. Married
  4. In a registered same-sex civil partnership
  5. Separated but legally married
  6. Divorced
  7. Widowed
  8. Separated from civil partner
  9. A former civil partner
  10. A surviving civil partner
  11. Living as a couple
- **hhtype_dv**: Composition of household.
  The variable hhtype_dv represents the composition of the household and is derived based on the Labour Force Survey (LFS) classification scheme. It categorizes households by counting the number of adults and children present, without taking into account which adults are responsible for the children. Unlike the original LFS classification, this version considers all individuals who are married, cohabiting, or in a same-sex relationship as couples.
  1. 1 male, aged 65+, no children
  2. 1 female, age 60+, no children
  3. 1 adult under pensionable age, no children
  4. 1 adult, 1 child
  5. 1 adult, 2 or more children
  6. Couple both under pensionable age, no children
  7. Couple 1 or more over pensionable age, no children
  8. Couple with 1 child
  9. Couple with 2 children
  10. Couple with 3 children
  11. 2 adults, not a couple, both under pensionable age, no children
  12. 2 adults, not a couple, one or more over pensionable age, no children
  13. 2 adults, not a couple, 1 or more children
  14. 3 or more adults, no children, incl. at least one couple
  15. 3 or more adults, 1-2 children, incl. at least one couple
  16. 3 or more adults, > 2 children, incl. at least one couple
  17. 3 or more adults, no children, excl. any couples
  18. 3 or more adults, 1 or more children, excl. any couples
- **Istrtdaty**: Individual interview start date (year).
  Provides (non-imputed) information on the start of individual interview (year).
- **ncrr1:** Non co-resident relationship.
  This variable captures whether a participant is in a steady romantic relationship with someone who does not live in the same household (response scale: yes; no). It is derived from responses to the question: ‘Do you have a steady relationship with someone you are not living with here, whom you think of as your 'partner'? Please include your spouse or partner if you are not currently living with them.’ This variable was only asked of respondents who did not have a spouse or partner living in their household (i.e., LiveSp ≠ 1 and LiveWith ≠ 1), ensuring that it identifies non-cohabiting partnerships only.

1. **Used variables for analyses (see Measures and Analytical Strategy in Method)***Note:* Only the content levels are listed, and missing value markers are omitted.
2. **SOEP Variables:**

- **pequiv/p11101**: Overall life satisfaction.
  This variable captures respondents’ global evaluation of their life satisfaction at the time of the interview. It is collected annually from all household members aged 16 and older (survey question: ‘How satisfied are you with your life, all things considered?’; response scale: 0 to 10, with 0 = completely dissatisfied and 10 = completely satisfied). The item is part of the SOEP’s core well-being module and is available in the pl-file. The harmonized variable p11101 is derived directly from plh0182.
- **pequiv/d11102ll**: This variable indicates the gender of each individual as recorded in the SOEP. Gender is treated as time-invariant and is only assessed once during panel enrollment; the value is then carried forward for all subsequent waves. The variable is derived directly from the variable sex in the ppfad file. Gender was recorded at the time of first participation and is missing only for a small number of cases where no information was provided and no inference was possible.
  1. Male
  2. Female
  3. Does not apply
- **pequiv/d11101**: Age.
  This variable indicates the age of each individual in years at the time of the interview. It is calculated based on the recorded year of birth and the year of data collection. Age is derived by subtracting the individual’s year of birth (GEBJAHR) from the survey year (SYEAR).
- **pequiv/ijob1**: Wages/salary from main Job.
  This variable represents the gross annual income from an individual’s main job, recorded for all household members aged 16 and older. It is derived by multiplying the number of months during which income was received in the previous year by the average monthly income from the main job, as reported by the participant. If information on the number of income-receiving months was missing, the sample mean of that variable was imputed. The resulting income values are reported in current-year Euros and range from 0 to 999,999. The variable is drawn from the PKAL file and reflects pre-tax gross earnings. If participants do not receive income from secondary employment, the corresponding variable is coded as 'Does not apply'.
- **pequiv/ijob2**: Income from secondary employment.
  This variable captures the gross annual income from secondary employment for all individuals aged 16 and older in the household. It is calculated by multiplying the number of months in the previous year during which income from secondary employment was received by the average monthly income amount. In cases where information on the number of months was missing, the sample mean for that variable was imputed. Reported in current-year Euros, the values for this variable range from 0 to 999,999. The data are drawn from the PKAL file. If participants do not receive income from secondary employment, the corresponding variable is coded as 'Does not apply'.
- **pequiv/iself**: Income from self-employment.
  This variable reflects the gross annual income from self-employment for all individuals aged 16 years and older in the household. It is calculated as the product of the number of months in the previous year during which income from self-employment was received and the average monthly income. If the number of income-receiving months was missing, the sample mean of that variable was imputed. The resulting income values are expressed in current-year Euros and range from 0 to 999,999. The data stem from the PKAL file. If participants do not receive income from secondary employment, the corresponding variable is coded as 'Does not apply'.
- **Netincome**: Monthly net income of participants.
  This variable was created by aggregating income from main employment (ijob1), secondary employment (ijob2), and self-employment (iself), and converting the total into a monthly amount. To ensure comparability across data sets, net income was z-standardized within each data set prior to inclusion in the analyses (see Method section for further details).
- **pequiv/d11108**: Education level.
  This variable indicates the highest educational attainment of individuals aged 16 and older. Based on detailed information from the SOEP education modules, this variable was collapsed into three harmonized categories to enable cross-national comparability: (1) Less than High School, including lower secondary education (e.g., Hauptschule), intermediate secondary education (e.g., Realschule), or no school-leaving certificate; (2) High School, including qualifications such as the Abitur (general qualification for university entrance), Fachhochschulreife (entrance qualification for universities of applied sciences), vocational training, and specialized vocational schools; and (3) More than High School, which includes education at institutions such as universities, universities of applied sciences, civil service academies, and health sector schools. The variable is coded as -1 in cases of item non-response.

**UKHLS Variables:**

- **sclfsato**: Satisfaction with life overall.
  This variable measures overall life satisfaction as reported by participants in the UKHLS. It is based on the question: *“Your life overall,”* and participants are asked to rate their satisfaction with life as a whole. The variable captures subjective well-being and is included in the indresp file. Respondents provide their answer on a scale from 1 to 7 (see below), where higher values indicate greater life satisfaction. The question was administered via face-to-face interviews (with self-completion), as well as telephone or web-based modes. For harmonization across the UKHLS and SOEP datasets, life satisfaction was z-standardized within each data set before being used in the analyses (see Method section for details).
  1. Completely dissatisfied
  2. Mostly dissatisfied
  3. Somewhat dissatisfied
  4. Neither satisfied nor dissatisfied
  5. Somewhat satisfied
  6. Mostly satisfied
  7. Completely satisfied
- **sex_dv**: Sex, derived.
  This variable indicates the respondent's sex and is derived from all available information across waves to ensure longitudinal consistency. It is coded as 1 for male, 2 for female, and 0 if the data are inconsistent and the respondent’s forename does not clearly indicate a gender. This derived variable is based on w_sex.
  1. Inconsistent
  2. Male
  3. Female
- **dvage**: Age from date of birth.
  represents the respondent’s age at their last birthday, calculated from their exact date of birth and the interview date (numeric value >= 0). If the date of birth is missing, estimated or previously reported age information is used, prioritizing Wave 6 data and cross-wave verified sources. For rare cases with no birth data available, the originally reported age (AGEIF) is used as a fallback.
- **fimnlabnet_dv**: Amount income component 1: net labor income.
  This variable represents net labor income, derived as the sum of three components (numeric value >= 0): net usual pay (w_paynu_dv), net self-employment income (w_seearnnet_dv), and net pay from a second job (w_j2paynet_dv). It provides an aggregated measure of individual earnings from all forms of labor. For harmonization across the UKHLS and SOEP data sets, net income was z-standardized within each data set before being used in the analyses (see Method section for details).
- **hiqual_dv**: This variable represents the highest educational or vocational qualification ever reported by a respondent. It is a derived and continuously updated variable based on multiple data sources, including initial conditions, later interviews, and cross-wave information. The categorization ranges from university degrees to vocational certificates and "no qualification," using a harmonized scheme that incorporates both UKHLS and BHPS respondents, with additional considerations for immigrant and youth subsamples.
  1. Degree (recoded as More than High School)
  2. Other higher degree (recoded as More than High School)
  3. A-level etc (recoded as High School)
  4. GCSE etc (recoded as High School)
  5. Other qualification (excluded from the analyses due to insufficient comparability across data sets)
  6. No qualification (Less than High School)

**Table S1**

*Model descriptions and formulas*

| Model | Model Formula | Description | Results |
| --- | --- | --- | --- |
| ***Research Question 1*** |  |  |  |
| Overall Average Model | life_satisfaction_z ~ moved_in_timepoint + (1 \| pid) | Random intercept model (person level) examining the overall average effect of moving in with a partner on life satisfaction. | Pooled data: Table S2  UKHLS: Table S7  SOEP: Table S8 |
| ***Research Question 2*** |  |  |  |
| Getting Married Interaction Model | Life_satisfaction_z ~ moved_in_timepoint * married + (1 \| pid) | Random intercept model (person level) analyzing the interaction between marital status and the year in which the move took place. | Pooled data:  Table S5  UKHLS: Table S7  SOEP: Table S8 |
| Getting Married x Epoch Interaction Model | life_satisfaction_z ~ moved_in_timepoint * married * epoch + (1 \| pid) | Random intercept model (person level) analyzing the interaction between marital status, the year in which the move took place, and timepoint effects of moving in together (epoch) on life satisfaction in Germany. | SOEP:  Table S8 |
| ***Research Question 3*** |  |  |  |
| Age Interaction Model | life_satisfaction_z ~ moved_in_timepoint * age_z + (1 \| pid) | Random intercept model (person level) assessing whether the effect of moving in with a partner on life satisfaction varies by age. | Pooled data:  Table S4  UKHLS: Table S7  SOEP: Table S8 |
| Gender Interaction Model | life_satisfaction_z ~ moved_in_timepoint * gender + (1 \| pid) | Random intercept model (person level) examining if the association between moving in with a partner and life satisfaction differs by gender. | Pooled data:  Table S4  UKHLS: Table S7  SOEP: Table S8 |
| Net Income Interaction Model | life_satisfaction_z ~ netincome_z * moved_in_timepoint + (1 \| pid) | Random intercept model (person level) investigating whether the effect of moving in with a partner on life satisfaction is influenced by net income. | Pooled data:  Table S4  UKHLS: Table S7  SOEP: Table S8 |
| Educational Attainment Interaction Model | life_satisfaction_z ~ education_level * moved_in_timepoint + (1 \| pid) | Random intercept model testing if the effect of moving in with a partner on life satisfaction varies by educational attainment. | Pooled data:  Table S4  UKHLS: Table S7  SOEP: Table S8 |
| Country of Residence Interaction Model | life_satisfaction_z ~ moved_in_timepoint * study + (1 \| pid) | Random intercept model (person level) assessing differences in the effect of moving in with a partner on life satisfaction between the UK and Germany. | Pooled data:  Table S4  UKHLS: Table S7  SOEP: Table S8 |

*Note:* The model formulas are based on Wilkinson notation as implemented in the 'lme4' package. Life_satisfaction_z = life satisfaction Z-score values sample mean standardized; moved_in_timepoint = categorical variable indicating time of moving in with a partner from 2 years before to 2 years after the event; gettingmarried = binary indicator variable, whether or not a participant marries after moving in with their partner during the observation period; epoch_z = Year (Z-score value) in SOEP data, where the move took place (between 1984–2019); age_z = continuous variable (Z-score value) indicating age; gender = man (reference) vs. woman; netincome_z = continuous variable (Z-score value) indicating net income calculated from sum of first and second job as well as self-employment; education_level = categorical variable with levels (1) More than High School, (2) High School, (3) Less than High School; study = UKHLS (reference) vs. SOEP; Overall models for Research Questions 2 and 3 were also run, including all interaction terms and main effects, controlling for socio-demographic factors.

**Table S2**

*Estimated mean differences in life satisfaction two years before to two years after moving in with a new partner (fixed effects analysis) using merged data (UKHLS & SOEP)*

|  | | **Life Satisfaction (Z Scores)** | | | | |  |
| --- | --- | --- | --- | --- | --- | --- | --- |
| *Predictors* | | *Estimates* | | *CI* | | *p* |  |
| **Fixed Effects** | |  | |  | |  |  |
| 0 (Moving in time point, intercept or reference) | | 0.15 | | 0.09 – 0.21 | | **<0.001** |  |
| -2 (Two years prior moving in together) | | -0.35 | | -0.41 – -0.29 | | **<0.001** |  |
| -1 (One year prior to moving in together) | | -0.16 | | -0.22 – -0.10 | | **<0.001** |  |
| 1 (One year after to moving in together) | | -0.07 | | -0.14 – -0.01 | | **0.041** |  |
| 2 (Two years after moving in together) | | -0.09 | | -0.17 – -0.02 | | **0.015** |  |
|  | |  | |  | |  |  |
| **Random Effects** |  | |  | |  | | |
| σ^2^ | 0.52 | |  | |  | | |
| τ_00_ | 0.46 | |  | |  | | |
| ICC | 0.47 | |  | |  | | |
| *N* | | 1,103 | |  | |  |  |
| Observations | | 4,641 | |  | |  |  |

**Table S3**

*Estimated mean differences in life satisfaction two years after moving in with a new partner (fixed effects analysis) using merged data (UKHLS & SOEP) with stepwise inclusion of covariates*

| **Model (Covariates)** | **AIC** | **BIC** | **Time Point ‘1’** | **Time Point ‘2’** |
| --- | --- | --- | --- | --- |
| Baseline (no covariates) | 11889.24 | 11934.33 | -0.07, ***p*= .042** | -0.09, ***p*= .015** |
| + Gender | 11895.24 | 11946.78 | -0.07, ***p*= .042** | -0.09, ***p*= .015** |
| + Age | 11893.22 | 11951.19 | -0.06, n.s. | -0.08, ***p*= .036** |
| + Income | 9201.74 | 9263.78 | -0.09, ***p*= .031** | -0.07, n.s. |
| + Education | 9052.28 | 9126.54 | -0.09, ***p*= .025** | -0.07, n.s. |
| + Country | 9057.50 | 9137.94 | -0.09, ***p* = .025** | -0.07, n.s. |

*Note.* The estimated values for time point ‘-2’ are consistently around -0.35 (all *p*s < .001) and for time point ‘-1’ are consistently around -0.16 SD (all *p*s < .001) in all models.

**Table S4**

*Estimated mean differences in life satisfaction two years before to two years after moving in with a new partner conditioned by potential sociodemographic moderators (linear mixed model) using merged data (UKHLS & SOEP)*

|  | **Life Satisfaction (Z Scores)** | | |
| --- | --- | --- | --- |
| *Parameters* | *Estimates* | *CI* | *p* |
| **Fixed Effects** |  |  |  |
| 0 (Moving in time point, Intercept) | 0.04 | -0.22 – 0.29 | 0.775 |
| -2 (Two years prior moving in together) | -0.22 | -0.49 – 0.05 | 0.105 |
| -1 (One year prior moving in together) | -0.21 | -0.48 – 0.06 | 0.131 |
| 1 (One year after moving in together) | -0.17 | -0.50 – 0.15 | 0.299 |
| 2 (Two years after moving in together) | -0.23 | -0.58 – 0.12 | 0.199 |
| Married: Getting married (Not getting married as reference) | 0.07 | -0.09 – 0.24 | 0.398 |
| Age (Z-scores) | -0.06 | -0.15 – 0.03 | 0.183 |
| Gender: Woman (Man as reference) | -0.01 | -0.15 – 0.12 | 0.840 |
| Net income (Z-scores) | 0.02 | -0.03 – 0.07 | 0.436 |
| Educational attainment: High School (less than High School as reference) | 0.11 | -0.15 – 0.37 | 0.402 |
| Educational Attainment: More than High School (Less than High School as reference) | 0.25 | -0.03 – 0.52 | 0.075 |
| Country of residence: UK (Germany as reference) | -0.06 | -0.24 – 0.12 | 0.508 |
| **Interactions** |  |  |  |
| -2 * Married: Getting married | -0.04 | -0.22 – 0.15 | 0.689 |
| -1 * Married: Getting married | -0.05 | -0.23 – 0.13 | 0.588 |
| 1 * Married: Getting married | 0.15 | -0.06 – 0.36 | 0.156 |
| 2 * Married: Getting married | 0.06 | -0.16 – 0.28 | 0.604 |
| -2 * Age (Z-scores) | -0.15 | -0.25 – -0.05 | **0.002** |
| -1 * Age (Z-scores) | -0.07 | -0.17 – 0.03 | 0.146 |
| 1 * Age (Z-scores) | 0.00 | -0.11 – 0.11 | 0.984 |
| 2 * Age: (Z-scores) | -0.02 | -0.14 – 0.10 | 0.770 |
| -2 * Gender: Woman | 0.05 | -0.09 – 0.19 | 0.511 |
| -1 * Gender: Woman | 0.09 | -0.05 – 0.24 | 0.195 |
| 1 * Gender: Woman | 0.09 | -0.07 – 0.26 | 0.278 |
| 2 * Gender: Woman | 0.06 | -0.12 – 0.25 | 0.517 |
| -2 * Net income (Z-scores) | 0.11 | 0.03 – 0.19 | 0.009 |
| -1 * Net income (Z-scores) | 0.14 | 0.06 – 0.22 | **<0.001** |
| 1 * Net income (Z-scores) | 0.06 | -0.02 – 0.14 | 0.153 |
| 2 * Net income (Z-scores) | 0.07 | -0.02 – 0.16 | 0.109 |
| -2 * Education: High School | -0.19 | -0.46 – 0.09 | 0.185 |
| -1 * Education: High School | -0.02 | -0.29 – 0.26 | 0.914 |
| 1 * Education: High School | -0.03 | -0.36 – 0.31 | 0.881 |
| 2 * Education: High School | 0.11 | -0.25 – 0.46 | 0.554 |
| -2 * Education: More than High School | -0.27 | -0.57 – 0.03 | 0.075 |
| -1 * Education: More than High School | -0.03 | -0.33 – 0.27 | 0.857 |
| 1 * Education: More than High School | -0.06 | -0.42 – 0.29 | 0.727 |
| 2 * Education: More than High School | 0.00 | -0.38 – 0.38 | 0.998 |
| -2 * Country of residence: UK | 0.17 | -0.02 – 0.37 | 0.078 |
| -1 * Country of residence: UK | 0.10 | -0.10 – 0.29 | 0.320 |
| 1 * Country of residence: UK | 0.16 | -0.07 – 0.39 | 0.179 |
| 2 * Country of residence: UK | 0.18 | -0.09 – 0.45 | 0.189 |
|  |  |  |  |
| **Random Effects** |  |  |  |
| σ^2^ | 0.50 |  |  |
| τ_00_ | 0.37 |  |  |
| ICC | 0.42 |  |  |
| *N* | 976 |  |  |
| Observations | 3,606 |  |  |

**Table S5**

*Estimated mean differences in life satisfaction when moving in and marrying vs. not marrying (linear mixed models) using merged data (UKHLS & SOEP)*

|  | **Life Satisfaction (Z Scores)** | | |
| --- | --- | --- | --- |
| *Predictors* | *Estimates* | *CI* | *p* |
| **Fixed Effects** |  |  |  |
| 0 (Moving in time point, Intercept) | 0.06 | -0.11 – 0.22 | 0.507 |
| -2 (Two years prior moving in together) | -0.32 | -0.40 – -0.25 | **<0.001** |
| -1 (One year prior moving in together) | -0.15 | -0.23 – -0.07 | **<0.001** |
| 1 (One year after moving in together) | -0.13 | -0.22 – -0.04 | **0.004** |
| 2 (Two years after moving in together) | -0.09 | -0.20 – 0.01 | 0.076 |
| Married: Getting married (Not getting married as reference) | 0.09 | -0.07 – 0.26 | 0.281 |
| Age (Z-scores) | -0.12 | -0.19 – -0.06 | **<0.001** |
| Gender: Woman (Man as reference) | 0.03 | -0.06 – 0.13 | 0.462 |
| Net income (Z-scores) | 0.08 | 0.04 – 0.12 | **<0.001** |
| Educational attainment: High School (less than High School as reference) | 0.04 | -0.12 – 0.20 | 0.630 |
| Educational Attainment: More than High School (Less than High School as reference) | 0.16 | -0.01 – 0.33 | 0.071 |
| -2 * Married: Getting married | -0.06 | -0.25 – 0.12 | 0.485 |
| -1 * Married: Getting married | -0.08 | -0.26 – 0.10 | 0.380 |
| 1 * Married: Getting married | 0.13 | -0.08 – 0.34 | 0.216 |
| 2 * Married: Getting married | 0.04 | -0.18 – 0.26 | 0.736 |
| **Random Effects** |  |  |  |
| σ^2^ | 0.50 |  |  |
| τ_00_ | 0.37 |  |  |
| ICC | 0.42 |  |  |
| *N* | 976 |  |  |
| Observations | 3,606 |  |  |

*Note.* For time points "-2" and "-1": married = participants who married in the future (at time point "0", “1”, or “2”) vs. participants who did not marry in the future (at time point “0”, “1”, or "2").

**Table S6**

*Sensitivity Analysis: Estimated mean differences in life satisfaction when moving in and marrying in the same year vs. not marrying (linear mixed models) using merged data (UKHLS & SOEP)*

|  | **Life Satisfaction (Z Scores)** | | |
| --- | --- | --- | --- |
| *Predictors* | *Estimates* | *CI* | *p* |
| **Fixed Effects** |  |  |  |
| 0 (Moving in time point, Intercept) | 0.11 | 0.04 – 0.16 | **0.001** |
| -2 (Two years prior moving in together) | -0.26 | -0.33 – -0.21 | **<0.001** |
| -1 (One year prior moving in together) | -0.12 | -0.19 – -0.06 | **<0.001** |
| 1 (One year after moving in together) | -0.08 | -0.14 – 0.01 | 0.069 |
| 2 (Two years after moving in together) | -0.07 | -0.15 – 0.01 | 0.083 |
| Married: Getting married (Not getting married as reference) | 0.06 | -0.08 – 0.21 | 0.383 |
| -2 * Married: Getting married | -0.03 | -0.19 – 0.12 | 0.652 |
| -1 * Married: Getting married | -0.04 | -0.19 – 0.11 | 0.572 |
| 1 * Married: Getting married | 0.09 | -0.09 – 0.26 | 0.332 |
| 2 * Married: Getting married | 0.07 | -0.12 – 0.26 | 0.468 |
| **Random Effects** |  |  |  |
| σ^2^ | 0.52 |  |  |
| τ_00_ | 0.45 |  |  |
| ICC | 0.46 |  |  |
| *N* | 997 |  |  |
| Observations | 4,196 |  |  |

*Note.* For time points "-2" and "-1": married = participants who married in the future (at time point "0") vs. participants who did not marry in the future (at time point “0”). Participants who married at time point “1” or “2” were excluded from the analysis.

**Table S7**

*Estimated mean differences in life satisfaction two years before to two years after moving in with a new partner conditioned by potential sociodemographic moderators (linear mixed model) using UKHLS data*

|  | **Life Satisfaction (Z Scores)** | | |
| --- | --- | --- | --- |
| *Parameters* | *Estimates* | *CI* | *p* |
| **Fixed Effects** |  |  |  |
| 0 (Moving in time point, Intercept) | 0.14 | -0.15 – 0.42 | 0.359 |
| -2 (Two years prior moving in together) | -0.08 | -0.41 – 0.25 | 0.636 |
| -1 (One year prior moving in together) | -0.11 | -0.44 – 0.23 | 0.526 |
| 1 (One year after moving in together) | 0.34 | -0.10 – 0.78 | 0.126 |
| 2 (Two years after moving in together) | -0.42 | -1.04 – 0.20 | 0.184 |
| Married: Getting married (Not getting married as reference) | -0.09 | -0.57 – 0.39 | 0.716 |
| Age (Z-scores) | -0.08 | -0.28 – 0.12 | 0.439 |
| Gender: Woman (Man as reference) | -0.26 | -0.58 – 0.06 | 0.104 |
| Net income (Z-scores) | -0.08 | -0.22 – 0.05 | 0.214 |
| Educational attainment: High School (less than High School as reference) | 0.14 | -1.61 – 1.88 | 0.876 |
| Educational Attainment: More than High School (Less than High School as reference) | 0.27 | -0.07 – 0.61 | 0.126 |
| **Interactions** |  |  |  |
| -2 * Married: Getting married | -0.44 | -0.96 – 0.08 | 0.099 |
| -1 * Married: Getting married | -0.15 | -0.67 – 0.38 | 0.588 |
| 1 * Married: Getting married | 0.01 | -0.63 – 0.65 | 0.967 |
| 2 * Married: Getting married | 0.46 | -0.19 – 1.11 | 0.164 |
| -2 * Age (Z-scores) | -0.09 | -0.32 – 0.14 | 0.432 |
| -1 * Age (Z-scores) | 0.01 | -0.22 – 0.23 | 0.965 |
| 1 * Age (Z-scores) | -0.08 | -0.39 – 0.22 | 0.579 |
| 2 * Age (Z-scores) | 0.05 | -0.30 – 0.41 | 0.766 |
| -2 * Gender: Woman | 0.17 | -0.19 – 0.53 | 0.353 |
| -1 * Gender: Woman | 0.30 | -0.05 – 0.66 | 0.092 |
| 1 * Gender: Woman | -0.00 | -0.42 – 0.41 | 0.988 |
| 2 * Gender: Woman | 0.29 | -0.21 – 0.80 | 0.255 |
| -2 * Net income (Z-scores) | 0.05 | -0.14 – 0.25 | 0.576 |
| -1 * Net income (Z-scores) | 0.04 | -0.15 – 0.23 | 0.674 |
| 1 * Net income (Z-scores) | 0.12 | -0.09 – 0.33 | 0.265 |
| 2 * Net income (Z-scores) | -0.00 | -0.25 – 0.25 | 0.989 |
| -2 * Education: High School | 0.64 | -1.29 – 2.58 | 0.512 |
| -1 * Education: High School | -1.55 | -3.48 – 0.38 | 0.116 |
| 1 * Education: High School | 0.47 | -1.47 – 2.42 | 0.632 |
| 2 * Education: High School | 1.09 | -1.12 – 3.30 | 0.331 |
| -2 * Education: More than High School | -0.26 | -0.64 – 0.13 | 0.187 |
| -1 * Education: More than High School | -0.11 | -0.49 – 0.28 | 0.587 |
| 1 * Education: More than High School | -0.49 | -0.98 – -0.01 | **0.047** |
| 2 * Education: More than High School | 0.27 | -0.34 – 0.88 | 0.389 |
|  |  |  |  |
| **Random Effects** |  |  |  |
| σ^2^ | 0.46 |  |  |
| τ_00_ | 0.28 |  |  |
| ICC | 0.38 |  |  |
| N | 146 |  |  |
| Observations | 560 |  |  |

**Table S8**

*Estimated mean differences in life satisfaction two years before to two years after moving in with a new partner conditioned by potential sociodemographic moderators (linear mixed model) using SOEP data*

|  | **Life Satisfaction (Grand Mean Z Scores)** | | |
| --- | --- | --- | --- |
| *Parameters* | *Estimates* | *CI* | *p* |
| **Fixed Effects** |  |  |  |
| 0 (Moving in time point, Intercept) | 0.02 | -0.23 – 0.28 | 0.859 |
| -2 (Two years prior moving in together) | -0.24 | -0.52 – 0.03 | 0.086 |
| -1 (One year prior moving in together) | -0.19 | -0.46 – 0.09 | 0.192 |
| 1 (One year after moving in together) | -0.20 | -0.53 – 0.13 | 0.238 |
| 2 (Two years after moving in together) | -0.23 | -0.59 – 0.14 | 0.223 |
| Married: Getting married (Not getting married as reference) | 0.04 | -0.16 – 0.24 | 0.702 |
| Epoch (Z-Scores) | 0.06 | -0.02 – 0.14 | 0.133 |
| Age (Z-scores) | -0.06 | -0.17 – 0.04 | 0.217 |
| Gender: Woman (Man as reference) | 0.03 | -0.12 – 0.17 | 0.724 |
| Net income (Z-scores) | 0.03 | -0.03 – 0.09 | 0.344 |
| Educational attainment: High School (less than High School as reference) | 0.11 | -0.15 – 0.37 | 0.408 |
| Educational Attainment: More than High School (Less than High School as reference) | 0.23 | -0.06 – 0.51 | 0.120 |
| **Interactions** |  |  |  |
| -2 * Married: Getting married | 0.02 | -0.20 – 0.25 | 0.831 |
| -1 * Married: Getting married | 0.02 | -0.20 – 0.25 | 0.831 |
| 1 * Married: Getting married | 0.20 | -0.05 – 0.45 | 0.110 |
| 2 * Married: Getting married | -0.01 | -0.30 – 0.27 | 0.919 |
| -2 * Epoch (Z-Scores) | -0.06 | -0.15 – 0.04 | 0.235 |
| -1 * Epoch (Z-Scores) | -0.03 | -0.13 – 0.06 | 0.464 |
| 1 * Epoch (Z-Scores) | 0.01 | -0.10 – 0.12 | 0.830 |
| 2 * Epoch (Z-Scores) | 0.02 | -0.11 – 0.14 | 0.814 |
| Married: Getting married * Epoch (Z-Scores) | -0.15 | -0.33 – 0.03 | 0.100 |
| -2 * Age (Z-scores) | -0.17 | -0.28 – -0.06 | **0.003** |
| -1 * Age (Z-scores) | -0.08 | -0.19 – 0.03 | 0.131 |
| 1 * Age (Z-scores) | -0.01 | -0.13 – 0.12 | 0.918 |
| 2 * Age (Z-scores) | -0.01 | -0.15 – 0.12 | 0.857 |
| -2 * Gender: Woman | 0.02 | -0.14 – 0.17 | 0.820 |
| -1 * Gender: Woman | 0.06 | -0.10 – 0.21 | 0.470 |
| 1 * Gender: Woman | 0.09 | -0.09 – 0.28 | 0.313 |
| 2 * Gender: Woman | 0.03 | -0.17 – 0.23 | 0.763 |
| -2 * Net income (Z-scores) | 0.15 | 0.06 – 0.24 | **0.002** |
| -1 * Net income (Z-scores) | 0.17 | 0.08 – 0.26 | **<0.001** |
| 1 * Net income (Z-scores) | 0.05 | -0.04 – 0.15 | 0.278 |
| 2 * Net income (Z-scores) | 0.08 | -0.02 – 0.17 | 0.120 |
| -2 * Education: High School | -0.18 | -0.46 – 0.10 | 0.206 |
| -1 * Education: High School | -0.04 | -0.33 – 0.24 | 0.767 |
| 1 * Education: High School | -0.02 | -0.36 – 0.31 | 0.890 |
| 2 * Education: High School | 0.13 | -0.23 – 0.50 | 0.477 |
| -2 * Education: More than High School | -0.23 | -0.54 – 0.08 | 0.147 |
| -1 * Education: More than High School | -0.04 | -0.35 – 0.27 | 0.785 |
| 1 * Education: More than High School | 0.01 | -0.36 – 0.38 | 0.958 |
| 2 * Education: More than High School | 0.01 | -0.39 – 0.41 | 0.952 |
| -2 * Married: Getting married * Epoch (Z-Scores) | 0.03 | -0.17 – 0.23 | 0.739 |
| -1 * Married: Getting married * Epoch (Z-Scores) | 0.10 | -0.10 – 0.30 | 0.331 |
| 1 * Married: Getting married * Epoch (Z-Scores) | 0.05 | -0.18 – 0.27 | 0.680 |
| 2 * Married: Getting married * Epoch (Z-Scores) | -0.05 | -0.31 – 0.20 | 0.690 |
|  |  |  |  |
| **Random Effects** |  |  |  |
| σ^2^ | 0.50 |  |  |
| τ_00_ | 0.38 |  |  |
| ICC | 0.43 |  |  |
| N | 825 |  |  |
| Observations | 3,028 |  |  |
